# Poisons and Poisoners

**Published:** 1891-10-03

**Authors:** 


### Poisons and Poisoners.

### III.—ANTIMONY—DR. PRITCHARD.

The last public execution in Scotland, witnessed by a hundred thousand persons, of whom there was hardly one who did not regard the criminal with abhorrence, was that of Dr. Pritchard, convicted of murdering his wife and mother-in-law with antimony. Some pity may be felt for a man who in a fit of passion strikes a fatal blow. He may be mad with anger, he has usually had provocation; but Pritchard had killed a woman who had always been a gentle and loving wife with cool and prolonged cruelty, and watched her dying by inches for months without compunction.

Edward William Pritchard was an Englishman by birth, but having married the daughter of a Glasgow silk merchant he settled in that town; and being a man of considerable attainments and pleasant manners he soon became popular. His family life seemed happy enough, his relations with his wife's family intimate and cordial; and it was only at the trial that it came out that he had promised to marry a servant girl in his house after his wife should die. Mrs. Pritchard had never been a strong woman, but about the end of October, 1864, her delicacy increased to decided invalidism. She grew pale, and her strength was reduced by continuous attacks of sickness. After the first attack of this nature she went to see her parents, who had now retired from business and gone to live in Edinburgh. She remained there for some time, and when she came home about Christmas she had regained her usual health. Within a week the old symptoms returned, and in the beginning of February she had such a violent attack of cramp that Dr. Cowan, a second cousin, was called in; and thinking that she was merely in a low state of health, prescribed champagne, with ice to soothe the irritability of the stomach, and injections of beef tea by way of nutriment. The treatment seemed to do no good, and another physician, Dr. Gairdner, was consulted. He was, as he afterwards said, "very much puzzled with the case," for he saw nothing in Mrs. Pritchard's condition to account for the constant vomiting, and he noted with curiosity the mental excitement from which she appeared to be suffering, and a peculiar spasm which contracted her hands. He ordered the stimulants to be discontinued, and next day found that the patient's condition had improved. Having to go to a consultation at some distance, he wrote to Dr. Pritchard to ask how his wife was, and was told by him that she was better, though not only was the statement false, but Dr. Pritchard was at this very time telling others that his wife was suffering from gastric fever.

About this time, February 10th, 1865, Mrs. Pritchard's mother, Mrs. Taylor, came to nurse her daughter. Though seventy years of age, she enjoyed good health, save for occasional neuralgic headaches. To relieve these she used Battley's sedative, a bottle of which she bought while staying at Dr. Pritchard's. After taking a dose of this she was very sick, as she had been after partaking of some tapioca, prepared especially for Mrs. Pritchard. Tapioca taken from the same package was afterwards found to contain antimony, while the bottle of sedative, though proved to be pure when bought, contained both antimony and aconite. On February 28th Mrs. Taylor, who had taken a dose of the sedative, became first sick, then comatose, and died in three hours. A neighbouring practitioner of good repute, Dr. Paterson, was called in to attend Mrs. Taylor. He expressed to Dr. Pritchard the opinion that she was suffering from narcotic poisoning. Dr. Pritchard replied that his mother-in-law was in the habit of using Battley's sedative, that she had lately bought a half-pound bottle of it, and that he had no doubt "she had taken a good swig of it." Dr. Paterson was asked to fill up the certificate of death, but declined, and in the note of refusal which he addressed to the registrar, he said "the death was certainly sudden, unexpected, and to me mysterious."

While he was with Mrs. Taylor, Dr. Paterson's attention had been arrested by the appearance of Mrs. Pritchard. "She seemed," he said in his examination, "to be exceedingly weak and exhausted. Her features were sharp and thin, with a high hectic flush on her cheeks, and her voice was very weak and exhausted." At first he was inclined to accept her husband's explanation of gastric fever, but he could not overcome the notion, which came to him while he was in her presence, that she was suffering from "the depressing influence of antimony."

He did not express his suspicions, a fact which was afterwards severely commented on; and even when, on March 2nd, Dr. Pritchard met him in the street and asked him to call on his wife—even then he said nothing, though he believed her to be suffering from poisoning by antimony, and prescribed accordingly. His defence was that he was called in only as a consultant to a patient who had already a doctor in the house; that, therefore, he had no right to return as if he were the physician in attendance, and that it would not have been safe for him to have said to Dr. Pritchard that his wife was being poisoned by antimony. The presiding judge, Lord Inglis, condemned this bowing to medical etiquette in no measured terms. The counsel for the defence, on the other hand, saw no harm in this, but tried to discredit Dr. Paterson's evidence by remarking on the animus he seemed to feel against the prisoner. It may well be that when the event justified the suspicions he had entertained Dr. Paterson's manner was distinctly hostile; it is likely enough, too, that he regretted that he had not taken measures to save the hapless victim from her fate. But, placed as he was, any medical man can easily understand the dilemma in which he found himself. To begin with, one may dislike a man, and even distrust him, without believing him to be capable of killing a wife with whom he lived in apparent amity; and, moreover, to have brought an accusation of attempted poisoning against Dr. Pritchard might have resulted in his being involved in a libel action, while the poor woman's doom might have been postponed, but hardly averted. Nor is it possible to ignore the fact that Dr. Paterson, like many other people, might, when the trial came on, regard as a firm conviction what at the time was only a vague suspicion. In any case, though we may not justify his action, one cannot but sympathise with his predicament.

After Mrs. Taylor's death Mrs. Pritchard's illness increased. Her husband seemed not only good, but tender. Her meals passed through his hands before they were sent to her—and they were sent through Mary McLeod, the servant-girl whom he had promised to marry when his wife died. He prepared sick-room dainties for her, he poured out her wine, and always after she had partaken of the food there came a return of the horrible sickness that was wearing out her life. At last, on March 17th, the end came. Mrs. Pritchard became delirious, spoke wildly of her mother and her children, and suffered from that cramp in the hands which Dr. Gairdner had previously noticed. Her supper was given her by her husband, and during the night another attack set in. A mustard poultice was applied, but at half-past one in the morning she was dead, her husband—the man who for five months had been killing her—lying by her side.

The deaths of mother and daughter following each other so quickly aroused suspicion. Three days after his wife's death Dr. Pritchard was arrested, and not only was Mrs. Pritchard's body subjected to chemical analysis, but Mrs. Taylor's was exhumed. In both bodies antimony was found, while in the mother's were traces of aconite as well. In neither was there any organic disease to account for death if no poison had been used. Experiments on animals showed that the Battley's solution and tapioca found in the house were not pure as when they were bought, while large and continuous purchases of antimony by Dr. Pritchard between September, 1864, and March, 1865, were proved by inquiry into the books of several druggists with whom he had dealt. So plain was it that Mrs. Taylor and Mrs. Pritchard had been poisoned that the prisoner's counsel tried to save his client only by throwing the onus of the crime on Mary M'Leod, who had more than sufficient interest in her mistress's death. But the crime was not the crime of an ignorant girl of seventeen. It wanted a skilled hand to choose a poison like antimony—it is arsenic, strychnia, or prussic acid that the vulgar choose—and to administer it in doses not sufficient to kill directly, but to reduce strength by repeated fits of sickness till exhaustion brought death. There was not a doubt in the public mind that the jury's verdict of guilty was well deserved. After his conviction Pritchard tried to implicate Mary M'Leod in his crime, but his statement won little credence and no sympathy. At last he confessed his guilt, saying that he had been actuated by "a species of terrible madness."

